# Biocompatibility testing of composite biomaterial designed for a new petal valve construction for pulsatile ventricular assist device

**DOI:** 10.1007/s10856-021-06576-w

**Published:** 2021-08-30

**Authors:** Roman Major, Maciej Gawlikowski, Hanna Plutecka, Marcin Surmiak, Marcin Kot, Marcin Dyner, Juergen M. Lackner, Boguslaw Major

**Affiliations:** 1 Institute of Metallurgy and Materials Science, Polish Academy of Sciences, Reymonta Str. 25, Cracow, Poland; 2 Foundation for Cardiac Surgery Development, Artificial Heart Laboratory, Wolnosci Str. 345, Zabrze, Poland; 3 Department of Biosensors and Processing of Biomedical Signals, Faculty of Biomedical Engineering, Silesian University of Technology, Roosevelt Str. 40, Zabrze, Poland; 4 Department of Medicine, Jagiellonian University Medical College, Skawinska Str. 8, Cracow, Poland; 5 Faculty of Mechanical Engineering and Robotics, AGH University of Science and Technology, Mickiewicza Str. 30, Cracow, Poland; 6 Faculty of Science and Technology, Jan Dlugosz University in Czestochowa, CHIRMED, 13/15 Armii Krajowej Av, Czestochowa, Poland; 7 Joanneum Research Forschungsges.m.b.H., Institute of Surface Technologies and Photonics, Functional Surfaces, Leobner Str. 94, Niklasdorf, Austria

## Abstract

This paper presents the results of biocompatibility testing performed on several biomaterial variants for manufacturing a newly designed petal valve intended for use in a pulsatile ventricular assist device or blood pump. Both physical vapor deposition (PVD) and plasma-enhanced chemical vapor deposition (PECVD) were used to coat titanium-based substrates with hydrogenated tetrahedral amorphous carbon (ta-C:H) or amorphous hydrogenated carbon (a-C:H and a-C:H, N). Experiments were carried out using whole human blood under arterial shear stress conditions in a cone-plate analyzer (ap. 1800 1/s). In most cases, tested coatings showed good or very good haemocompatibility. Type a-C:H, N coating proved to be superior in terms of activation, risk of aggregation, and the effects of generating microparticles of apoptotic origin, and also demonstrated excellent mechanical properties. Therefore, a-C:H, N coatings were selected for further in vivo studies. In vivo animal studies were carried out according to the ISO 10993 standard. Intradermal reactivity was assessed in three rabbits and sub-acute toxicity and local effects after implantation were examined in 12 rabbits. Based on postmortem examination, no organ failure or wound tissue damage occurred during the required period of observation. In summary, our investigations demonstrated high biocompatibility of the biomaterials in relation to thrombogenicity, toxicity, and wound healing. Prototypes of the petal valves were manufactured and mounted on the pulsatile ventricular assist device. Hydrodynamic features and impact on red blood cells (hemolysis) as well as coagulation (systemic thrombogenicity) were assessed in whole blood.

## Highlights

- Thin coatings.
- Biocompatibility.
- Haemocompatibility.
- In vio test.
- Intradermal reactivity.
- Sub-acute toxicity.

## Introduction

Heart valves are regulatory elements of the circulatory system responsible for ensuring proper blood flow through the heart. Dysfunction of any of these four valves can lead to bacterial myocarditis and even, heart failure [1]. Chronic and untreated valve incompetence leads to hemodynamic decompensation and heart insufficiency. The disorder can occur in the aortic valve (AoV), one of two arterial valves in the heart.

The AoV prevents blood in the aorta from flowing backwards into the left ventricle. A properly functioning AoV opens during contraction of the ventricles, allowing blood to flow from the left ventricle to the aorta, and closes diastole, to prevent retrogression. When the AoV does not close completely, blood accumulates in the left ventricle, which can overload the chamber and progressively impair function. This also increases the size of the heart. One example of a heart valve defect is constriction of the AoV and narrowing of the left arterial outlet, known as AoV stenosis, which causes pressure to build up in the left ventricle and decreased coronary circulation. Moreover, shear stress due to blood flow causes segmentation of von Willebrand factor [2, 3]. This reduction in AoV outlet area or stenosis impedes blood flow from the left ventricle to aorta. Narrowing of the AoV can continue for many years as the impeded outflow of blood from the left ventricle forces the heart to do more work, eventually leading to cardiac hypertrophy.

The initial period of the disease is usually asymptomatic. However, the thickened myocardium has a greater need for oxygen and nutrients. When coronary circulation does not provide enough blood, even to normal coronary arteries, angina arises. An enlarged heart will also be highly susceptible to ischemic damage; therefore, patients with cardiac hypertrophy are at much higher risk of mortality from extensive infarction.

Based on the rate of progression of aortic stenosis, we can classify aortic regurgitation into acute or chronic. Aortic regurgitation is chronic when receding blood leads to an increase in the volume of the left ventricle, volume overload, and compensatory muscular hypertrophy. In this case, it is not always necessary for a set of characteristic symptoms to appear. However, as time passes, the heart gradually grows bigger, and the contractility of the left ventricle decreases, a patient may start to exhibit signs of respiratory and cardiovascular failure. In acute aortic regurgitation, blood flow disturbances appear quickly.

A heart defect characterized by narrowing of the AoV is a chronic disease that can progress over several years without any symptoms. When symptoms do appear, the risk of sudden cardiac death increases significantly. Apart from valvuloplasty, another common way to restore normal heart valve function is by replacing it with a new valve. This could be a mechanical or biologic heart-valve prosthesis. Artificial valves have been implanted in patients since the early 1960s. At present, the number of heart valve transplantations per annum around the world has reached 300 thousand. However, surgery can pose additional risks, particularly for elderly patients suffering numerous comorbidities, which has led to the development of the transcatheter aortic valve implantation technique [4].

For several mechanical valves, design-related features are responsible for their improved thromboresistance [5]. However, it remains unclear whether material-related features provide a practical level of thromboresistance to mechanical valves. The authoors studied the effect of a bileaflet valve made of poly(ether ether ketone) (PEEK) with a poly(2-methacryloyloxyethyl phosphorylcholine) (PMPC)-grafted surface (PEEK-g-PMPC). It was mentioned that PMPC is a well-known thromboresistant polymeric material. A short-term (<26 h) porcine AoV replacement model using neither an anticoagulant nor an antiplatelet agent showed that the PEEK-g-PMPC valve opened and closed normally with an allowable transvalvular gradient.

From the perspective of the health and life of a patient, some important aspects of implanted valves include: optimal surgical outcome, biocompatibility of the implant material, resistance of both the biomaterial and valve structure to biodegradation, and adequate blood flow through the valve under working conditions in the human body. Furthermore, the implanted heart valve must not become damaged once implanted, as mechanical failure could lead to a drastic deterioration of health or death of the patient. Moreover, any allergies, inflammation, or infection caused by the valve material may interfere with hemostasis and effectiveness of the disease cure, and sometimes, necessitating reoperation. Multiple surgeries dramatically increase the associated risks.

This study aims to minimize the risk of life-threatening thrombo-emboli formation in petal valve designed to work in a pulsatile heart assist devices by redesigning a biomimetic heart valve. Heart prostheses often support patients with end-stage heart failure awaiting full heart transplant or on rare occasions, act as a bridge to myocardial recovery. In future, they could be combined with gene or stem-cells therapy to treat myocardial infarction. Heart valve prosthesis development must consider, and indeed prevent, several fluid dynamics phenomena such as cavitation and turbulent flow, as well as high shear stress during backflow through the narrow disc-ring gap. Mechanical heart valves can cause platelet activation, which is the first stage of thrombosis. Thus, to ensure optimal blood flow, further research on heart valve designs is still needed with a particular focus on hemo-biofunctional coatings, testing, and phantom models.

## Materials and methods

### Surface modification

Coating of titanium-based substrates was performed by physical vapor deposition and plasma-enhanced chemical vapor deposition (PECVD). A more detailed description is presented elsewhere [6]. Briefly, substrates were ultrasonically cleaned in alcohol and acetone to remove any contaminants on the surface. All subsequent processing was performed under cleanroom conditions to prevent dust deposits on surfaces.

Substrates were places in a vacuum deposition chamber (Leybold Oerlikon, Germany) in vertical position at a distance of ~80 mm from the magnetron sputtering cathodes and the chamber was evacuated to a pressure of 2 × 10^−5^ mbar. Prior to film deposition, a final cleaning and activation by ion etching were carried out by applying Ar-O_2_ plasma using a linear anode-layer ion source (ALS 340, Veeco, US). Film deposition was performed using different techniques: PECVD-based deposition using the anode-layer source to achieve hydrogenated tetrahedral amorphous carbon coatings (ta-C:H) in an acetylene atmosphere with partial addition of argon; Magnetron sputtering from pure pyrolytic carbon target materials (Schunk, Austria) in a mixed atmosphere of acetylene and argon to achieve amorphous hydrogenated carbon films (a-C:H); Additional mixing of nitrogen instead of argon in the same atmosphere to deposit nitrogen-containing hydrogenated amorphous carbon films (a-C:H, N) (Table 1).

**Table 1:** Deposition parameters and surface energy of coatings for heart valve

Composition						Thickness	Deposition method	Atmosphere	Rough-ness	Contact angle	Surface energy		
a-C:H	a-C:H:N	a-C:N	ta-C:H	ta-C:H:N	Si-a-C:H	[nm]		[sccm]	Ra [nm]		Polar	Disperse	Total
			X			92.2	Ionic source	20.0 C_2_H_2_		79.9	3.5	40.0.	43.4
			X			48.0	Ionic source	20.0 C_2_H_2_		78.3	3.5	42.4	45.9
			X			67.4	Ionic source	20.0 C_2_H_2_		79.7	3.3.	41.4	44.6
			X			90.8	Ionic source	5.0 Ar + 15.0 C_2_H_2_		84.0	2.1	40.8	43.0
X						94.7	Sputter	24.0 Ar + 6.0 C_2_H_2_	14.7	60.82	9.68	46.74	56.43
	X					103.8	Sputter	24.0 Ar + 4.5 C_2_H_2_ + 1.5 N_2_	14.2	58.53	10.17	48.82	58.99
	X					103.1	Sputter	24.0 Ar + 3.0 C_2_H_2_ + 3.0 N_2_	12.7	57.83	11.55	45.54	57.09
	X					95.2	Sputter	24.0 Ar + 1.5 C_2_H_2_ + 4.5 N_2_	24.0	53.03	13.70	46.55	60.25
		X				95.2	Sputter	24.0 Ar + 6 N_2_	19.0	45.90	17.14	47.45	64.59
			X			94.7	Ionic source	20.0 C_2_H_2_	13.0	41.87	20.94	43.6	64.54
				X		110.2	ionic source	17.5 C_2_H_2_ + 2.5 N_2_	23.3	25.37	30.09	41.61	71.70
				X		100.3	Ionic source	15.0 C_2_H_2_ + 5.0 N_2_	12.7	32.63	24.61	45.87	70.49
				X		82.7	Ionic source	12.5 C_2_H_2_ + 7.5 N_2_	14.3	38.80	25.99	36.90	62.89
					X	124.3	Sputter	28.2 Ar + 1.8 C_2_H_2_	16.3	27.80	27.07	45.35	72.42
					X	15.0	Sputter	28.2 Ar + 1.8 C_2_H_2_	41.3	22.93	29.66	44.17	73.83
					X	127.6	Sputter	27.0 Ar + 3.0 C_2_H_2_	19.2	19.37	29.47	46.89	76.36
					X	15.0	Sputter	27.0 Ar + 3.0 C_2_H_2_	38.3	25.97	29.98	41.36	71.33
					X	123.5	Sputter	24.0 Ar + 6.0 C_2_H_2_	26.2	24.35	27.62	47.07	74,.69
					X	15.0	Sputter	24.0. Ar + 6.0 C_2_H_2_	27.7	23.33	30.11	13.05	73.16
					X	118.5	Sputter	18.0 Ar + 12.0 C_2_H_2_	14.0	54.33	11.98	49.66	61.64
					X	15.0	Sputter	18.0 Ar + 12.0 C_2_H_2_	52.7	51.17	14.28	47.68	61.96

All gases were pure (N5.0) and provided by Linde Gas (Austria). Various durations of deposition resulted in different thicknesses. Coating parameters are presented in Table 1.

### Mechanical testing

Indentation tests were carried using 0.5 and 1 mN loads and a Berkovich indenter tip. Loading and unloading were performed at a speed of 1 and 2 mN/min, respectively, with a dwell time at maximum load of 5 s. Additional indentation tests were performed with Rockwell C geometry and rounding radius of 200 μm. Scratch tests were performed up to the maximum load of the 20 N indenter.

### Hemocompatibility assessment

To assess hemocompatibility, whole human venous blood samples were collected. P-selectin and fibrinogen receptor GP IIb-IIIa (PAC-1) were selected as platelet activation markers. Positive and negative controls were used to determine the number of aggregates [7–9]. Negative controls were obtained from human blood samples taken on sodium citrate to prevent clotting. Positive controls were prepared by mixing blood with adenosine diphosphate (ADP) to a final concentration of 20 mM. Polyurethane (PU) and polystyrene (PS) were used as substrate controls.

All samples influence the activation of PAC-1 and Selectin P antigens in a similar way, which can be measured as the percentage of blood platelets remaining in the blood after shear stress conditions compared to the percentage of platelets expressing P-selectin and PAC-1.

The enzyme-linked immunosorbent assay was performed to determine the number of platelet-derived microparticles in the blood after direct contact between materials and blood plasma samples (Zymuphen MP-TF, Hyphen Biomed, France). Microparticles are immobilized by annexin V and thrombogenic plasma activity associated with the presence of platelet microparticles was measured [10–13].

### In vivo examination

In vivo tests were carried out according to the ISO 10993 standard with regards to sub-acute toxicity (point 11), local effects after implantation (point 6), and intradermal reactivity (point 10). All animal experiments were approved by the local Bioethics Commission [14–24].

Tests were carried out on 12 male white New Zealand rabbits from the same litter aged 5–6 months with a body weight of 2.5–3.5 kg. Animals were fed complete food for rabbits ad libitum and provided with constant access to drinking water. One month before the experiment, animals were dewormed with 1% ivermectin (0.3 mg/kg; Vetos Farma, Poland) and vaccinated against the plague and myxomatosis (Pestorin Mormyx, Bioveta, Czech Republic). A 21-day quarantine was carried out before the observation of rabbits in their permanent breeding place. Feeding was stopped 12–18 h prior to the procedure but free access to water was still provided.

#### Course of intradermal reactivity experiment

**Fig. 1:**
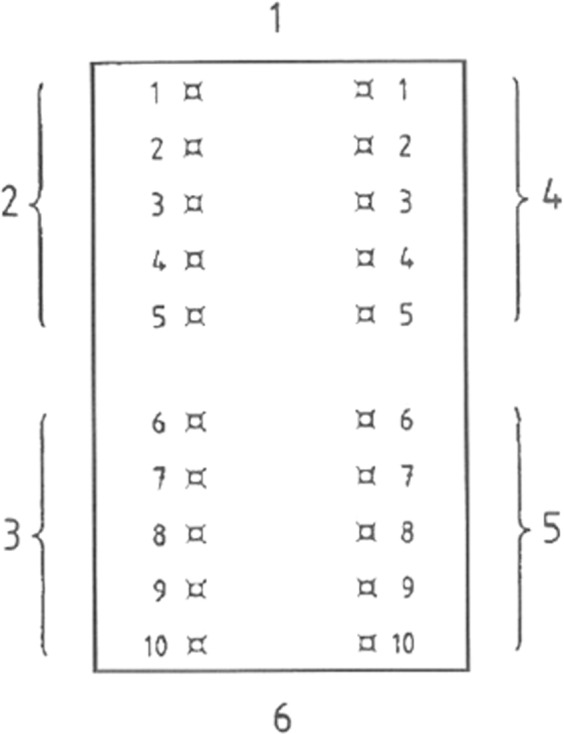
Intradermal reactivity and localization of injection points (according to ISO 10993-10). 1—head-side of animal, 2—injection of extract in polar solvent, 3—injection of pure polar solvent, 4—injection of extract in non-polar solvent, 5—injection of pure non-polar solvent, 6—tail-side of the animal

Intradermal reactivity experiments were performed in three rabbits, as previously described [25, 26]. No anesthesia was used. Animals were placed in the abdominal position and hair on the back was cut with a 50+ blade to a length of about 0.5 mm from the front edge of the shoulder to the ischial tumor and over the entire width of the ridge. Skin was washed three times with disinfectant (Skinsept, Ecolab, Germany). Ten intradermal injections were made on the right and left side the ridge and 0.2 ml of test material were implanted in a polar (0.9% NaCl) or non-polar (sesame oil) solvent, as shown in Fig. 1. Prior to implantation, all liquids were sterilized by radiation (25 kGy).

After injection, animals were observed at 24, 48, and 72 h. Erythema and edema were assessed separately. At the end of the experiment, all animals were transferred to a rabbit farm and allowed to enter the breeding stock.

#### Course of sub-acute toxicity and local effects after implantation experiments

Sub-acute toxicity and local effects after implantation were assessed, according to a previously described protocol [26]. Twelve rabbits were used: six in the experimental group and six in the control group. Surgeries was performed under anesthesia. Briefly, implants with a diameter of 15 mm and a thickness of 1.5 mm were placed under the skin over the longest dorsal muscle. After 28 days of observation, animals were euthanized, and tissues were removed for further testing. The control group underwent same procedure without introducing an implant.

##### Anesthesia

Anesthesia included premedication and induction of anesthesia with Xylazine HCl at an intramuscular dose of 10–15 mg/kg (2% Xylapan, Vetoquinol, Poland) and ketamine at an intramuscular dose of 2–5 mg/kg (10% ketamine, Biowet Poland). General anesthesia was performed depending on the effects of the above-mentioned preparations, successively adding 1/5–1/4 of the initial dose. Before surgery, a 20GA cannula (Venflon 1.0, BD) was inserted into the right or left ear vein to ensure access to the blood vessel.

##### Surgical procedure

Each implantation were performed by the same operator with extensive clinical experience under the same conditions and lasted about 25 min. Animals were placed in the abdominal position and a 10 cm-wide cut was made along the ridge from the shoulder to pelvis using a 50+ blade machine for a length of about 0.5 mm. The cutting area was not shaved. The skin was washed three times with disinfectant (Skinsept, Ecolab Deutschland GmbH). The area of the wound was covered with a sterile surgical drape 75 × 90 cm with a 6 × 8 cm opening and a plaster (Euro Centrum, Poland). A transverse cut of 1.5 cm was made on the right and left side of the ridge just below the last rib and ~1 cm from the midline of the ridge. A pocket with a length of ~3–4 cm and a width of 1.5–2 cm was dissected from the wound into the subcutaneous tissue.

In the experimental group, a disc with a diameter of roughly 8–10 mm and a thickness of 1.5 mm was inserted into each pocket and the wound was sewn shut ~3–4 cm from the edge of the wound using a 3/0 polypropylene thread (DKO37PP, Yavo, Poland), maintaining a minimum of 2 cm between the edge of the implant and the edge of the wound. In the control group, an incision and pocket were made and secured with single suture.

Aluminum spray dressing was applied to the wound (Aluspray, MediVet, Poland) to prevent infection. After the procedure, 100.0 ml of 0.9% sodium chloride (NaCl, B. Braun, Poland) was subcutaneously administered. For 3 days, 0.5 mg/kg Meloxicam analgesics (5 mg/ml Melovem, Dopharma, The Netherlands) was intramuscularly injected. In addition, 5 mg/kg of antibiotic (10% Enrofloxacin, Drwalew, Poland) as subcutaneously injected for 5 days.

##### Euthanasia and collecting tissue samples

After 28 days, animals were euthanized. A two-stage euthanasia process was used. During the first stage, animals underwent general anesthesia, as above. After complete elimination of consciousness and sensation, animals were intravenously administered 100 mg/kg of pentobarbital sodium (Morbital, Biowet). After euthanasia, a postmortem examination of the implant area was carried out and tissue was collected for further examination. The following tissue samples were collected: skin over the implant with a minimum 1 cm margin, hang under the implant with a margin of at least 1 cm, the heart tip, left lower lobe, a fragment of the middle lobe, right and left kidneys, spleen, and right and left knee lymph node.

##### Histological and hematological examination

Samples were fixed in 4% formaldehyde for 48 h, and embedded in paraffin. Routine hematoxylin–eosin (H&E) staining and Masson’s trichrome staining were carried out. H&E provides an overall picture of the tissue structure, while Masson’s trichrome is used to observe specific tissue components including nerves, collagen, and myofibers.

To assess toxicity of the material, several morphological, biochemical, and coagulation tests were carried out on blood. Citrated whole blood morphology was assessed using the BC VET 2800 apparatus (Mindray). Biochemistry tests included alanine aminotransferase (ALT), aspartate aminotransferase (AST), glutamyl-transpeptidase, lactate dehydrogenase, alkaline phosphatase, as well as assessments of creatinine and total protein. The above parameters were measured in blood serum using a manual biochemistry analyzer (Rayto, Stamar). Coagulation was assessed based on activated partial thromboplastin time (APTT) and prothrombin time using the STart 4 Hemostasis Analyzer (Stago).

## Analysis of results

### Mechanical analysis

**Fig. 2:**
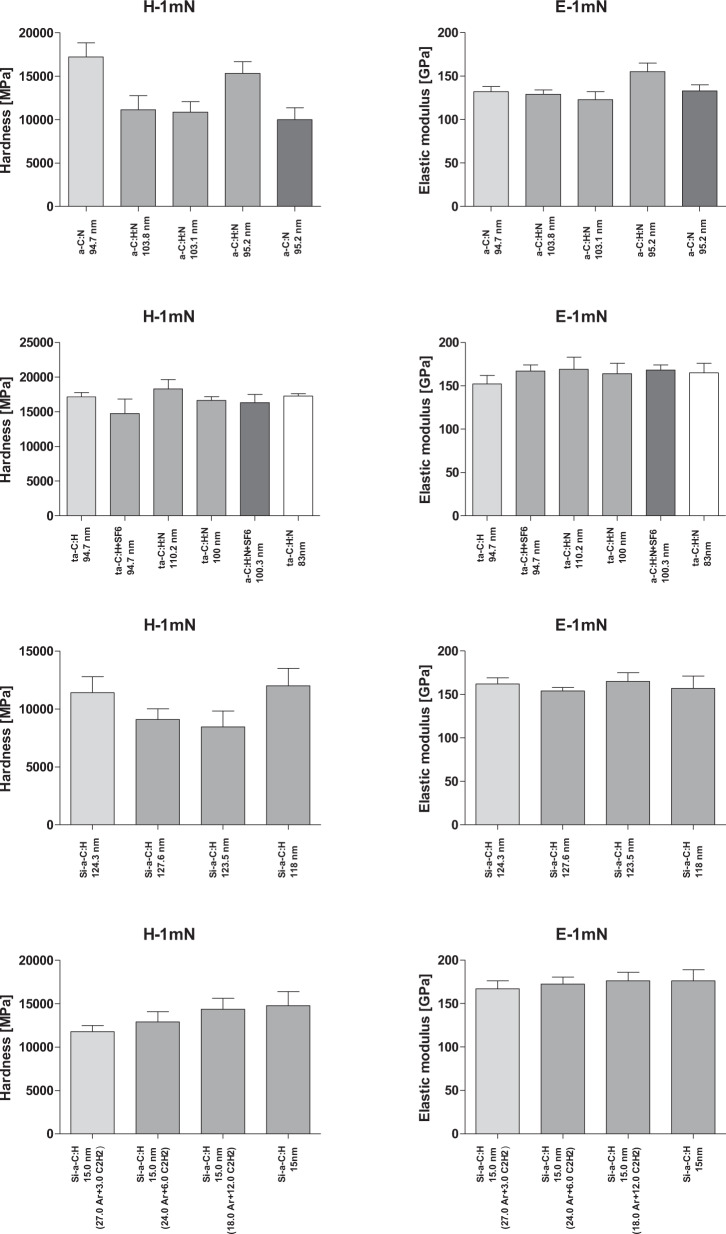
Comparison of the measured values of modulus of elasticity of coatings described in Table 1

Hardness of the α-C:H coating was highest (17.2 GPa) and introducing nitrogen into the structure, i.e., α-C:H, N coatings, significantly reduced the hardness (Fig. 2). The lowest hardness value was obtained for α-C:N with no hydrogen. Hardness of the B372_9 coating, which is the hardest of the α-C:H, N coatings, produced by the ratio of C2H2 to N2 of 1.5:4.5, were surprising.

Applying an additional thin PTFE coating (via fluoridation) only slightly decreased the measured hardness value and differences in hardness and modulus of elasticity were within the limits of measurement scattering. Hardness of coatings produced at the intermediate C2H2 to N2 gas ratios of 28.2:1.8 and 27:3 were significantly lower than those produced at gas ratios of 28.2:1.8 and 18:12. Moreover, differences in the modulus of elasticity were within the limits of measurement scatter. Coating hardness increased as the ratio of C2H2 to N2 increased from 28.2:1.8 to 18:12. Differences in elastic modulus were small, although a slight increase in the measured mean value was observed.

**Fig. 3:**
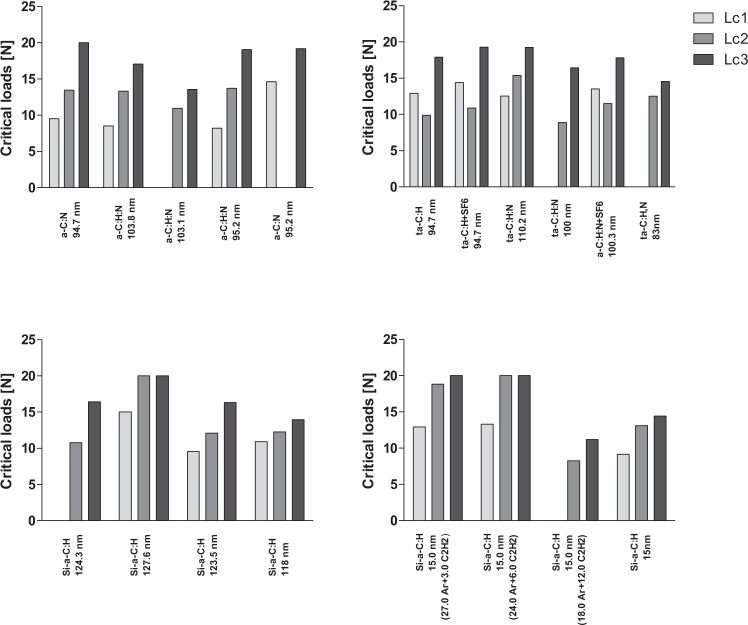
Critical load values of LC1–3 of the coatings described in Table 1

Initially, fine wiping was visible for some coatings under an optical microscope (LC1). Other important parameters were also compared including the load causing cracks on the surface of the coating (LC2), chipping, and loss of adhesion to the substrate (LC3), as shown in Fig. 3.

### Hemocompatibility results

**Fig. 4:**
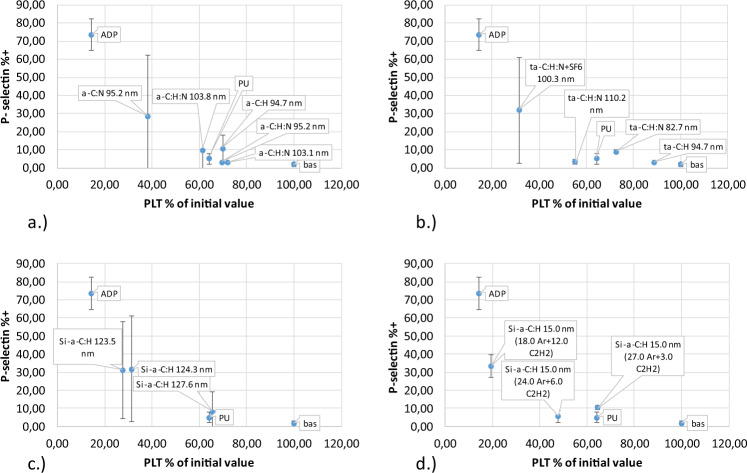
Activation of P-selectin receptor caused by direct contact with material (Table 1) under high shear condition: **a** a-C:H, a-C:H,N **b** a-C:H, a-C:H + SF6 (fluoridation), a-C:H,N, a-C:H,N + SF6 (fluoridation), **c** Si/a-C:H, **d** Si/a-C:H 15 nm

**Fig. 5:**
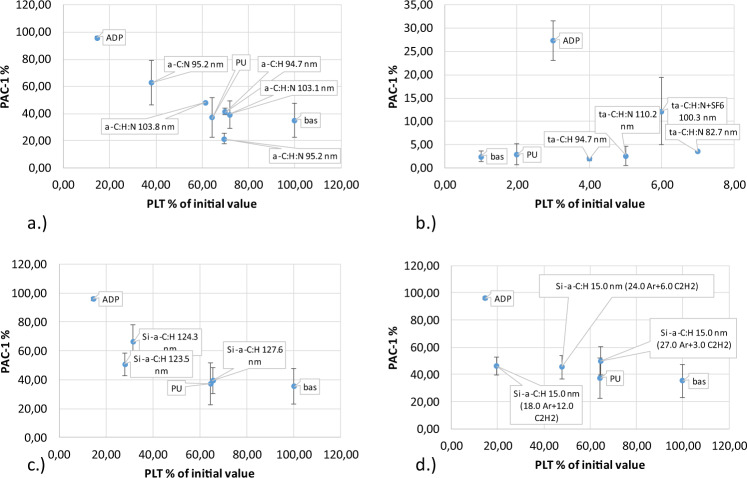
Activation of platelet IIb/IIIa receptor caused by direct contact with the tested material (Table 1) under high shear condition: **a** a-C:H, a-C:H,N, **b** a-C:H, a-C:H + SF6 (fluoridation), a-C:H,N, a-C:H,N + SF6 (fluoridation), **c** Si/a-C:H, **d** Si/a-C:H 15 nm

Activation was measured as the percentage of blood platelets remaining in the blood after the shear test compared to the percentage of platelets expressing P-selectin (Fig. 4 and PAC-1, Fig. 5). Analysis of P-selectin activation shows that most coatings had little effect on activation processes, without using remaining platelets in the blood. The second IIb/IIIa receptor showed similar activation properties. Some coatings exceeded the acceptable value, for example coating a-C:H, N with a thickness of 95.2 nm, as shown in Table 1. These coatings were eliminated from further analyses.

**Fig. 6:**
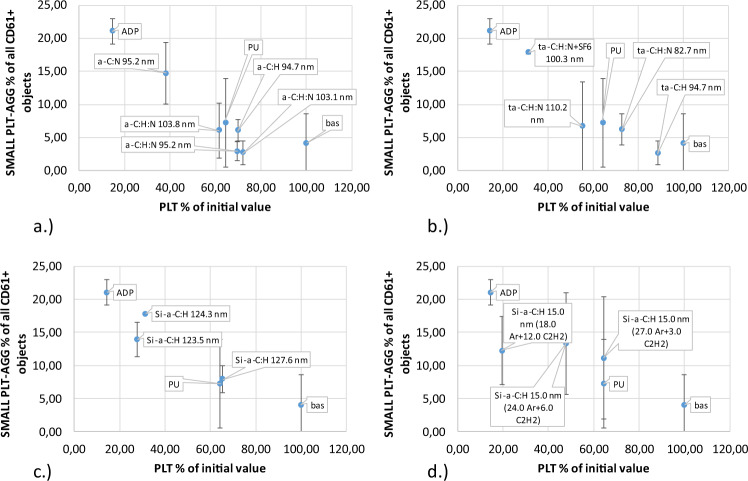
Small platelet aggregate caused by direct contact with material (Table 1) under high shear stress: **a** a-C:H, a-C:H,N, **b** a-C:H, a-C:H + SF6 (fluoridation), a-C:H,N, a-C:H,N + SF6 (fluoridation), **c** Si/a-C:H, **d** Si/a-C:H 15 nm

**Fig. 7:**
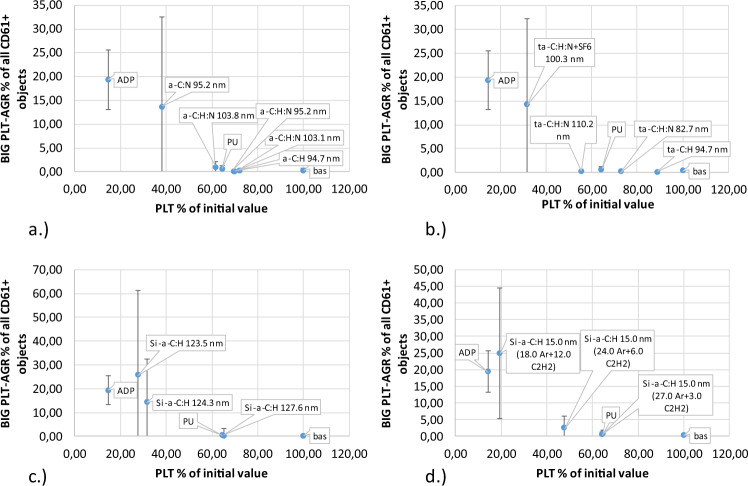
Large platelet aggregate caused by direct contact with material (Table 1) under high shear stress: **a** a-C:H, a-C:H,N, **b** a-C:H, a-C:H + SF6 (fluoridation), a-C:H,N, a-C:H,N + SF6 (fluoridation), **c** Si/a-C:H, **d** Si/a-C:H 15 nm

Throughout the activation process, platelet aggregation occurs when the artificial surface is in direct contact with blood flow under high shear conditions. Excessive platelet aggregation is an uncontrolled physiological mechanism that regulates platelet aggregation. Under physiological conditions, platelet adhesion is activated by direct contact with the artificial surface onto which platelets adhere. Platelet activation of releases substances that trigger a chain of biochemical reactions and as a consequence, blood clotting. Results of platelet aggregation after shearing were compared with all CD61-positive platelets. In this work we distinguish between small and large platelet aggregates. Small platelet aggregates are classified as two platelets grouped together (Fig. 6), whereas large platelet aggregates consist of more than two platelets (Fig. 7).

Direct contact of artificial surfaces with surrounding tissue, in this case of blood, can lead to degradation of the material substructure. Release of membrane proteins (MPs) from eukaryotic cells is a normal physiological process that occurs during maturation and aging of cells. Increased release of MPs may occur when cells are activated, for example by binding to complementary components or immune complexes, cytokines, chemokines, stress factors such as temperature, changes in osmotic pressure, voltage generated by blood flow through vessels, or factors leading to cell apoptosis.

**Fig. 8:**
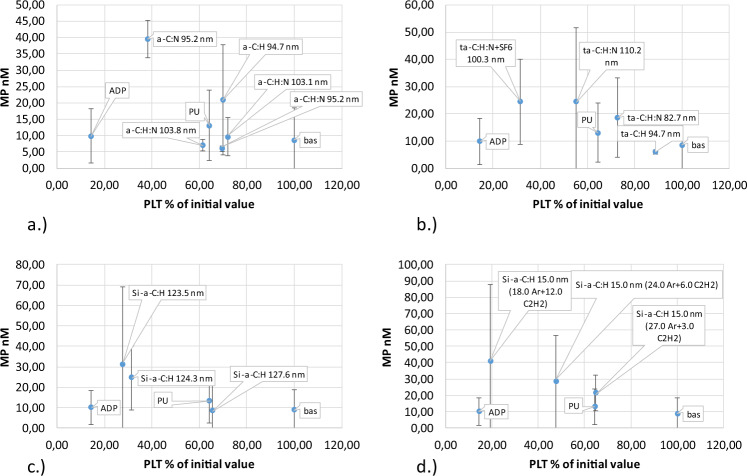
Platelet-derived microparticles created by direct contact with artificial surface (Table 1) under shear stress condition: **a** a-C:H, a-C:H,N, **b** a-C:H, a-C:H + SF6 (fluoridation), a-C:H,N, a-C:H,N + SF6 (fluoridation), **c** Si/a-C:H, **d** Si/a-C:H 15 nm

Coatings with the most favorable parameters for contact with blood were selected based on the concentration of microparticles. The results presented in Fig. 8 provide important information proving the lack of platelet-derived microparticles under dynamic conditions, despite full activation using ADP. Under static conditions, the concentration of microparticles does not increase due to ADP blood activation, probably owing to the rapid aggregation of platelets. Conditions of shear stress always lead to an increased microparticle concentration.

**Fig. 9:**
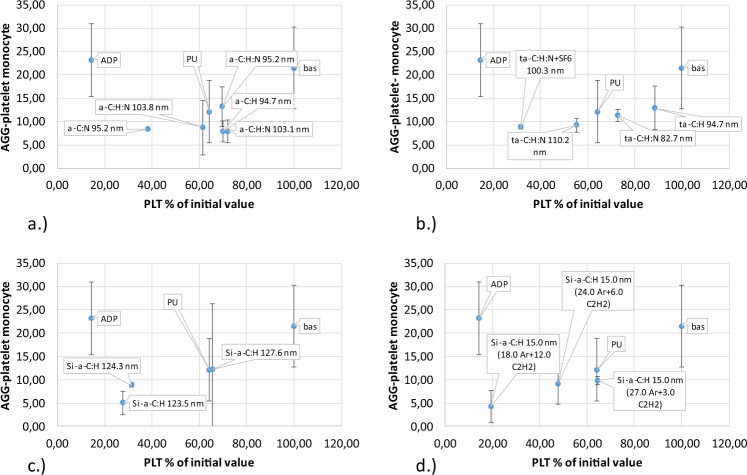
Platelet-monocyte aggregates created by direct contact with material (Table 1) under high shear condition: **a** a-C:H, a-C:H,N, **b** a-C:H, a-C:H + SF6 (fluoridation), a-C:H,N, a-C:H,N + SF6 (fluoridation), **c** Si/a-C:H, **d** Si/a-C:H 15 nm

Assessing the likelihood of creating monocyte-platelet aggregates is even more informative than the probability of creating microparticles. Interaction between leukocytes and platelets occurs only through the interaction of endothelial P-selectin and leukocyte PSG-1 glycoprotein. Specifically, monocytes are involved in the formation of leukocyte-platelet aggregates, which suggests that monocyte-platelet aggregation is a more sensitive test for platelet activation than P-selectin surface expression. Degranulation causes rapid loss of P-selectin. Moreover, the creation and detachment of the so-called microparticles plate (microplate) is important since these fragments possess antigens, CD61 and CD41, capable of forming a functionally efficient GPIIb/IIIa fibrinogen receptor. Platelet-monocyte aggregation as a function of the percentage of platelets is presented in Fig. 9.

Calculations show that a-C:H, N coatings with a thickness of 95.2 nm are characterized by the best hemocompatibility properties in direct contact with human blood and exhibit excellent mechanical properties. Based on the mechanical properties and hemocompatibility, this material was selected for in vivo studies.

### In vivo examination

#### Investigation of intradermal reactivity

**Fig. 10:**
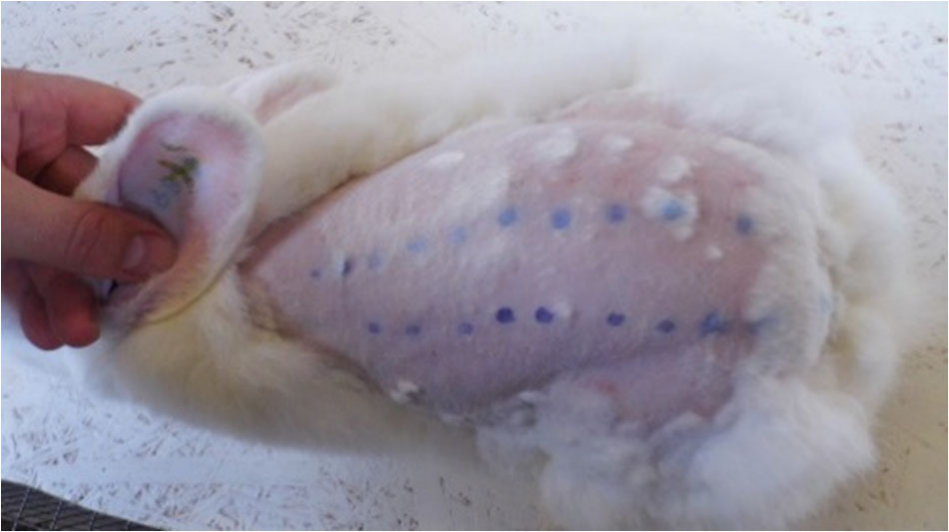
Representative image animal skin 72 h after injection

Figure 10 shows the animal skin 72 h after intradermal injection. Skin irritation was evaluated using intradermal reactivity tests according to PN EN ISO 10993-10 and the scale of the reaction is presented in Table 2.

**Table 2:** Scale of skin reaction in intradermal reactivity tests

Reaction	Irritation score
Erythema formation	
No erythema	0
Very slight erythema	1
Well defined erythema	2
Moderate erythema	3
Severe erythema (dark red)	4
Edema formation	
Lack of edema	0
Very weak swelling (barely perceptible)	1
Well defined erythema (edges of the area well defined by elevation)	2
Moderate edema (elevation of about 1 mm)	3
Painful swelling (elevation above 1 mm that goes beyond the area of exposure)	4
Maximum possible score	8

Animals did not show a significant degree of erythema and/or edema after intradermal injection of material extracts. The average irritation score induced by physiological saline and oil solution and extracts of material in these solvents was 0.13.

Test results showed that the material extracted with non-polar and polar solvents did not irritate the skin of rabbits during the 72 h observation period. Tested materials met requirements since the difference between the average test score and the average control score was <1.0 (=0.13), which is in accordance with the normative guidelines ISO 10993-10 and considered non-irritating. Thus, it can be concluded that the tested material does not induce intradermal reaction.

#### Sub-acute toxicity and local effects after implantation experiments

No significant differences in crucial parameters of blood morphology were found. Before implantation and before euthanasia, all examined parameters were in the physiological range in both the experimental and control group. In general, biochemical parameters revealed no relevant changes, however ALT in both groups was elevated. Moreover, total protein and AST were slightly under the normal range. Coagulation parameters were assessed only before euthanasia. No normal ranges of APTT were found in the rabbits, therefore empirical ranges were assumed based on the experimenters’ experience. Detailed results and ANOVA or Student *t* statistical test are presented in Table 3.

**Table 3:** Detailed results of morphology, biochemistry, and coagulation

Parameter	Research group		Control group		Normal range for rabbit
	Before implantation	Before euthanasia	Before implantation	Before euthanasia	
WBC	12.2 ± 6.2	11.7 ± 5.0	9.3 ± 2.9	10.8 ± 3.3	5.2–13.5
RBC	5.2 ± 0.3	5.8 ± 0.3	5.0 ± 0.4	6.0 ± 0.3	5.0–7.6
MCV	63.5 ± 2.5	64.3 ± 2.0	65.7 ± 2.9	64.0 ± 2.6	56.8–66.5
HGB	112.2 ± 5.8	121.3 ± 4.5	108.7 ± 8.3	122.4 ± 2.9	105–170
HCT	32.8 ± 1.6	37.5 ± 1.6	32.7 ± 1.4	38.4 ± 1.6	31.0–46.0
PLT	202 ± 74	291 ± 82	319 ± 66	364 ± 70	100–512
AST	22.3 ± 5.2	49.2 ± 17.7	23.4 ± 3.0	35.3 ± 3.3	42–98
Bilirubin	0.14 ± 0.07	0.07 ± 0.03	0.08 ± 0.02	0.07 ± 0.02	0.0–0.7
ALP	92.2 ± 21.1	68.4 ± 15.8	104.0 ± 18.0	71.1 ± 14.1	90–145
GGTP	7.14 ± 2.4	2.9 ± 2.4	8.0 ± 4.3	3.6 ± 2.9	<40^a^
LDH	96 ± 21	140 ± 47	69 ± 25	188 ± 68	<400^b^
Creatinine	1.18 ± 0.08	1.22 ± 0.09	1.23 ± 0.09	1.34 ± 0.13	0.5–2.7
Protein	41.2 ± 2.8	41.4 ± 1.3	40.0 ± 1.5	40.7 ± 1.3	60–83
APTT	–	14.3 ± 2.9	–	14.2 ± 3.8	26.0–36.0
PT	–	7.4 ± 1.4	–	6.5 ± 0.5	10.0–14.0

^a^No data for rabbit. The normal range for human was assumed

^b^Dependently on examination method and laboratory standard

Control measurements of body mass before implantation and before euthanasia reveal slight weight gain: +10% in the experimental group and +8% in the control group. Daily observation of animals was carried out and both post-operative wound healing and animal behavior were assessed.

**Fig. 11:**
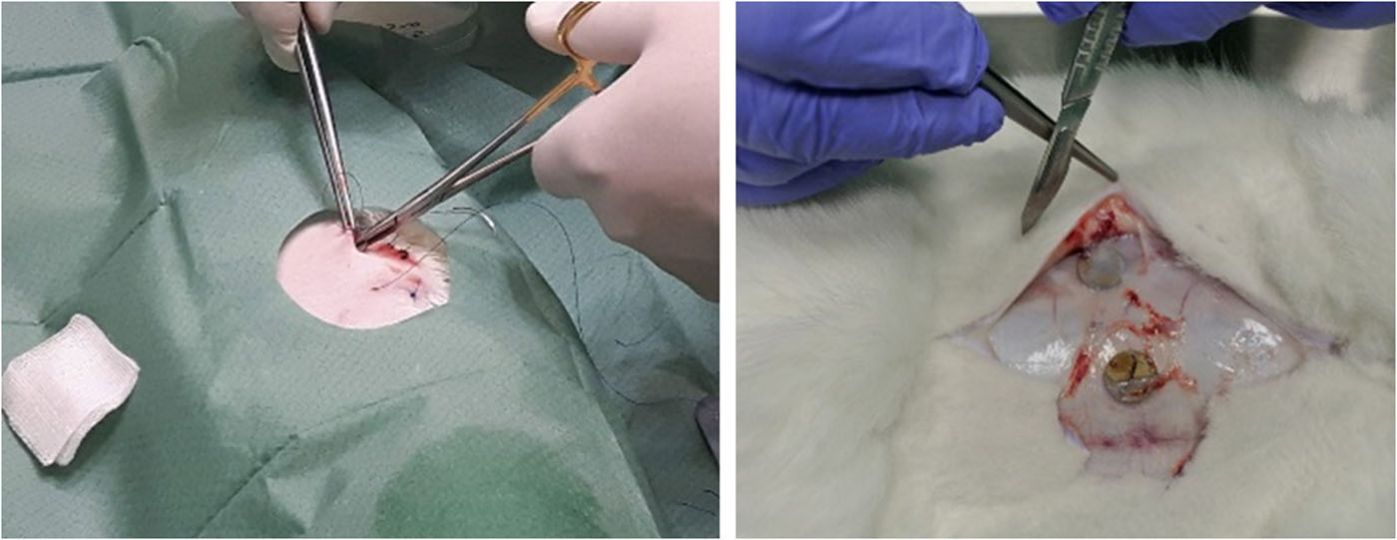
Photograph of minor ecchymosis in the experimental (left) and control group (right)

In five out of 12 animals, postmortem evaluation of the wounded area did not reveal any major or minor pathological lesions. In other animals, minor ecchymosis (Fig. 11) and inflammatory reactions were observed around the wound and scar tissue. Lesions were present both in the experimental and control group and therefore likely unrelated to presence of the biomaterial. In one animal, one iatrogenic complication occurred in the form of major abdominal inflammation.

Microscopic evaluation of tissue preparations revealed the following:

- No pathological lesions in cardiac tissue.
- Lack of toxic reaction nor other lesions in liver, spleen and thymus tissue.
- No symptoms of inflammation nor alveoli pathology in lung tissue (in five cases, minor intrapulmonary bleeding was observed as a result of tightness during euthanasia).
- In five cases, features of inflammation glomerulopathy were observed (two in the left and three in the right kidney) probably related to breeding specificity. In all other cases, no symptoms of toxic inflammation or neutrophilic infiltration were observed.

In conclusion, no symptoms of toxicity due to the biomaterial were encountered based on hematological and histological examination.

#### Post-operative complications

One complication was observed. On day 16 after the procedure, a focal purulent lesion of eczema, about 4 cm in diameter appeared on one rabbit at the height of the implant penetrating the dermis layer but not the subcutaneous tissue. A local dressing (Aluspray, MediVet, Poland) was applied for 6 days with an antibiotic cover. After 3 days, skin amines subsided. In the postmortem examination, healing after biting was visible but did not reaching the subcutaneous tissue.

#### Adverse events

Throughout the course of the experiment, most animals did not show any signs of adverse reaction. In one case, healing problems were observed. During the first week following implantation, slight inflammatory changes were observed at the implant site (left side of the spine). In general, no clinical changes were observed. All rabbits (except one) healed without complication. All wounds healed rapidly.

**Fig. 12:**
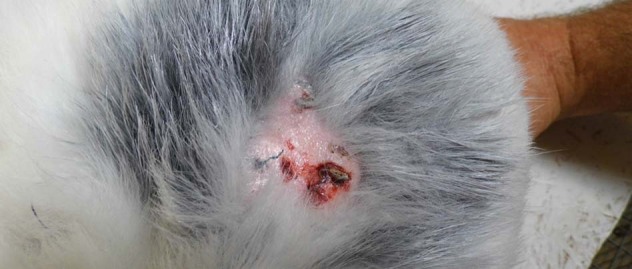
Skin lesion 16 days post-surgery (gray-colored hair was caused by aluminum spray used for wound protection)

In many cases, local lesions of varying intensity were observed in the immediate vicinity of the implant in the form of small bruises with no inflammatory features. This may be due to minor ischemia in the immediate vicinity of the implant as a result of mechanical irritation, as confirmed by histological examination (Fig. 12).

Following antibiotic therapy, changes were reversed and the wound completely healed. Assessment of weight gain did not show significant changes for most animals. There were no significant changes in whole blood morphology to suggest pathology. Blood biochemistry test results showed no changes before and after surgeries indicating no systemic effects as a result of the implanted material. In five out of 12 animals, sectional evaluations showed no changes at the site of implantation and no organ or tissue pathologies. In all other animals, changes were observed at the implant site, mainly ecchymosis and minor inflammatory changes. In one animal, severe inflammatory changes within the abdominal cavity were observed.

#### Histological evaluation

Histological evaluation of collected organ and tissue samples confirmed the lack of local reaction after implantation and no noticeable toxic effects due to the presence of the material. Pathological changes in one implant in the form of inflammatory glomerulopathy may be related to culture conditions. Observed changes in the pulmonary vasculature are likely related to the procedure itself or may be due to the tissue sectioning process. The lack of infectious lesions within skin margins and the reproducibility of histologic images of implant site margins suggest the veterinary surgeries were carried out correctly.

In summary, no local inflammatory reactions were observed at implant sites and there were no significant organ pathologies to indicate systemic toxicity 4 weeks post-implantation.

## Discussion

In this study, the surface of the petal valve was modified with selected coatings and the whole valve was reconstructed. The results confirm the innovative properties of the new ReligaHeart EXT ventricular assist device construction [27, 28] equipped with elastic valves: single-petal at the inlet and double-petal at the outlet with flushing channels. The composite design integrates the petals of the valves with the surrounding parts and ensures that the blood flows across the smooth surfaces of the petals. The valve petals were reinforced with a metallic frame to eliminate creep effects and problems associated with thermal and time stability.

Scaffolding and injection molding technologies for polymers were developed to produce a prototype of the valves for in vitro testing and in vivo testing on large animal models. A new precision machining technology was also developed to build the metal frames of the inlet and outlet valve rings. More specifically, a precision laser cutting technique was developed to cut the thin-walled metal nets that make up the frame for the inlet and outlet valve flaps. Following the laser cutting process, the metallic frames were tested and examined using advanced scanning electron microscopy (SEM) and transmission electron microscopy techniques. As an alternative to SEM, laser scanning confocal microscopy was used to examine the metal topography.

A series of titanium scaffolds was constructed for the development of biocompatible coatings using high-pressure injection methods. Elastic, biocompatible multilayer coatings based on TPU and Ti were developed and their microstructure, chemical composition, surface topography, wetting angle, adhesion of coatings, and mechanical properties were characterized.

Thin coatings were prepared from the following materials: Ti, acid-proof steel, PU, and PS-reference materials. The microstructure, chemical composition, surface topography, adhesion of coatings and mechanical properties were investigated. A total of 19 coatings were tested, divided into four groups: evolution of the hydrogenated carbon α-C:H coating to the nitrogen-rich α-C:N; coatings with additional fluoridation of the α-C:H and α-C:N coatings; coatings with silicon atoms with thicknesses of ~120 nm (group 3a) and 15 nm (group 3b), respectively.

Hemocompatibility tests were conducted under hydrodynamic conditions. The following tests were performed to measure the polymer properties: hysteresis loop method to determine fatigue properties, dynamical-mechanical thermal analysis in the range of −100 °C to +150 °C, biocorrosion/biodegradation tests, analysis of swelling of and adhesion to a metallic frame, contact angle measurements using the drop shape method, differential scanning calorimetry, rheological tests (ARES) performed at a range of temperature from −100 °C to +150 °C, viscosity measurements using a Brookfield viscometer, and a series of extrusion tests to evaluate the geometry and uniformity of thickness of extruded polymers.

In addition, fatigue properties were determined using the hysteresis loop method, as well as dynamical and mechanical thermal analysis from −100 °C to + 150 °C, measurement of temperature-dependent elastic properties (at 30, 50, 70, and 90 °C), and rheological measurements using the melt flow index (MFI).

Metal frames dedicated to flooding were prepared and a technology for sinking metallic part in a polymer sheath was developed. A prototype series of valve petals (mesh embedded in PU) were constructed. For the final design of the valves, equipment required for assembling the new valves in the ReligaHeart EXT ventricular assist device were developed. Current market analysis was extended to the cost of such equipment, including the valve testing station in hydrodynamic conditions and the visualization system.

Based on the analyses, materials for in vivo animal tests were elaborated to test the selected coatings in the animal model. Ready-to-use valves were prepared for performing blood tests in an artificial patient model.
